# Amino Acid Substitutions at the Major Insertion Loop of *Candida albicans* Sterol 14alpha-Demethylase Are Involved in Fluconazole Resistance

**DOI:** 10.1371/journal.pone.0021239

**Published:** 2011-06-16

**Authors:** Nidia Alvarez-Rueda, Audrey Fleury, Florent Morio, Fabrice Pagniez, Louis Gastinel, Patrice Le Pape

**Affiliations:** 1 Département de Parasitologie et de Mycologie Médicale, Université de Nantes, Nantes Atlantique Universités, EA1155 – IICiMed, Faculté de Pharmacie de Nantes, Nantes, France; 2 Laboratoire de Parasitologie-Mycologie, CHU de Nantes, Nantes, France; 3 Laboratoire de Pharmacologie des Immunosuppresseurs en Transplantation, INSERM UMR 850, Université de Limoges, Limoges, France; University of Minnesota, United States of America

## Abstract

**Background:**

In the fungal pathogen *Candida albicans,* amino acid substitutions of 14alpha-demethylase (CaErg11p, CaCYP51) are associated with azole antifungals resistance. This is an area of research which is very dynamic, since the stakes concern the screening of new antifungals which circumvent resistance. The impact of amino acid substitutions on azole interaction has been postulated by homology modeling in comparison to the crystal structure of *Mycobacterium tuberculosis* (MT-CYP51). Modeling of amino acid residues situated between positions 428 to 459 remains difficult to explain to date, because they are in a major insertion loop specifically present in fungal species.

**Methodology/Principal Finding:**

Fluconazole resistance of clinical isolates displaying Y447H and V456I novel CaErg11p substitutions confirmed *in vivo* in a murine model of disseminated candidiasis. Y447H and V456I implication into fluconazole resistance was then studied by site-directed mutagenesis of wild-type CaErg11p and by heterogeneously expression into the *Pichia pastoris* model. CLSI modified tests showed that V447H and V456I are responsible for an 8-fold increase in fluconazole MICs of *P. pastoris* mutants compared to the wild-type controls. Moreover, mutants showed a sustained capacity for producing ergosterol, even in the presence of fluconazole. Based on these biological results, we are the first to propose a hybrid homology structure-function model of Ca-CYP51 using 3 different homology modeling programs. The variable position of the protein insertion loop, using different liganded or non-liganded templates of recently solved CYP51 structures, suggests its inherent flexibility. Mapping of recognized azole-resistant substitutions indicated that the flexibility of this region is probably enhanced by the relatively high glycine content of the consensus.

**Conclusions/Significance:**

The results highlight the potential role of the insertion loop in azole resistance in the human pathogen *C. albicans*. This new data should be taken into consideration for future studies aimed at designing new antifungal agents, which circumvent azole resistance.

## Introduction

*Candida albicans* is an opportunistic fungal pathogen that causes severe blood and disseminated infections (BSIs). The incidence of these infections has markedly increased over the past decade, due to the increase of immunocompromised and neutropenic patients after organ transplantation, cancer therapy or AIDS [1]. In these populations, *C. albicans* is associated with a high mortality rate and the obvious economic consequences [2], [3].

Although some antifungal drugs are available (see review [4], [5]), fluconazole (FLC) is still considered the drug of choice to treat most *Candida* infections [6]–[8]. However, long-term exposure to FLC, as well as FLC underdosage during the empirical regimens, leads to an increased resistance phenomena [9]–[16]. Thus, the understanding of the molecular mechanisms underlying azole resistance in *C. albicans* is necessary in order to discover new antifungal agents that circumvent drug resistance [17], [18] and could guide the choice of the appropriate antifungal treatment at the onset of infection.

Among the molecular mechanisms involved in the resistance of *C. albicans* to azole antifungals we can distinguish the over-expression of the gene encoding efflux pumps CDR1, CDR2 and MDR1 [19]–[22] and/or upregulation or amino acid substitutions of lanosterol 14alpha-demethylase (CaErg11p, CaCYP51) [11], [12], [23]–[26]. CaErg11p participates in ergosterol biosynthesis, an essential requirement for yeast viability [27]. Mutations in the *CaErg11* gene can result in amino acid substitutions in the CaErg11p which lead to the decreased affinity of FLC for its target and can lead to toxic lanosterol accumulation [28]. Among the many CaErg11p substitutions described to date in *in vitro* azole-resistant clinical isolates of *C. albicans* (See review [29]), only a few of them have been implicated in resistance, using site-directed mutagenesis [12], [30]–[32]. These mutations are clustered into three hot spots, ranging from amino acids 105 to 165, 266 to 287 and 405 to 488 [13].

In a previous study, the *CaErg11* gene sequencing of 73 *C. albicans* clinical isolates displaying various levels of azole susceptibility, allowed us to identify two novel amino acid substitutions, Y447H and V456I, in two clinical isolates (CAAL61 and CAAL37 respectively) displaying a high-level of FLC resistance (64 µg/ml) [29]. Although V456I substitution has been previously mentioned in the literature by Sanglard D and coll. [33], its involvement in azole resistance has not been investigated so far. Both amino acid residues located in the third hot spot (amino acid residues 405 to 488) are conserved in various yeast and filamentous fungal species [29], [34]. Whereas, substitutions at these positions could be involved in azole resistance. However, the interaction of amino acid residues between positions 428 to 459 with FLC is still difficult to explain because of a major insertion loop specifically present in fungal species [35]. To the best of our knowledge, molecular information of this region is lacking, due to the absence of amino acid sequence homology between CaErg11p and bacterial- or trypanosomatid- CYP51 molecular models [34], [36]–[40]. Taken together, the involvement of both Y447H and V456I substitutions in FLC resistance remains difficult to solve using the current homology models of CaCYP51.

Site-directed mutagenesis of the *CaErg11* gene and its heterologous expression in the yeast *Saccharomyces cerevisiae* is the most commonly employed model to investigate the potential involvement of CaErg11p point substitutions on azole resistance [31], [41], [42]. More recently, *Pichia pastoris* has emerged as a practical expression system and is considered a viable alternative to bacterial and *S. cerevisiae* expression models [43]–[45]. *Pichia pastoris* has also been suggested as an efficient method for mutant library creation, based on the selection of single copy plasmid integrated transformants on low antibiotic concentrations [46]. Furthermore, the *P. pastoris* expression model has some advantages such as: (i) the repression of endogenous genes with low production levels of native proteins, (ii) the genetic stability of the *P. pastoris* transformants by gene integration, (iii) the capacity for eukaryotic post-translational modifications such as glycosylation and (iv) the possibility of a high production of mutated recombinant proteins.

In the present study, we introduced single mutations into the *CaErg11* gene by site-directed mutagenesis. We cloned the *CaErg11* gene and mutants into *P. pastoris* to study the contribution of Y447H and V456I amino acid substitutions in FLC resistance. We also investigated the effect of these Erg11p substitutions on ergosterol biosynthesis. Based on the recently published CYP51 ortholog sequence alignments and structures, we first proposed a hybrid model of CaCYP51 in order to localize amino acid substitutions in the major insertion loop and to explain their possible implication on azole resistance.

## Results

### Azole susceptibility of clinical isolates

Minimal inhibitory concentrations (MICs) of the CAAL37 and CAAL61 clinical isolates against FLC, ITC and VRC were determined *in vitro* (Table 1). The two isolates were resistant for FLC (MIC  = 64 µg/ml). The ITR and VRC MICs were changed in these isolates, although not reaching clinical breakpoints (CAAL37: 0.125 and 0.06 µg/ml respectively; CAAL61: 0.25 and 0.25 µg/ml respectively). FLC resistance was also confirmed *in vivo* in a murine model of disseminated candidiasis. The median survival of mice groups receiving *C. albicans* isolates CAAL37, CAAL61 and CAAL97 in the absence of drug treatment, were respectively 2, 3 and 4 days. Virulence of *C. albicans* was not significantly different between these drug-free groups (p = 0.102 between groups). Whereas 5 mg/kg of FLC had a significant survival effect on mice infected by the susceptible CAAL97 isolate (median survival of 13 days after FLC treatment), both CAAL37 and CAAL61 clinical isolates showed a drastically reduced FLC susceptibility *in vivo* (median survival of 3 days). The Kaplan-Meier survival plot clearly revealed a significant mortality disadvantage of CAAL37 and CAAL61 isolates after treatment with FLC compared to the CAAL97 isolate (p = 0.02 vs CAAL97) (Figure S1).

**Table 1:** Azole antifungal susceptibility and amino acid substitutions in CaErg11p for the clinical isolates of *C. albicans*.

Strain	Site of isolation	MIC (µg/ml)			Amino acid change in Erg11
		FLC	ITR	VRC	
CAAL37	Respiratory tract	>64	0.125	0.06	E266D, G464S, **V456I** ^h^, V488I
CAAL61	Mouth	>64	0.250	0.250	G307S, **Y447H**
CAAL97	ND	0.120	0.250	0.015	D116E, K128Y

h  =  heterozygous (i.e., mutation in a single allele).

### Construction of *P. pastoris* transformants expressing mutated and wild-type CaErg11p

The general strategy of mutagenesis into *P. pastoris* is shown in Figure S2 and Figure S3. A single colony of each *P. pastoris* mutants, wild-type and mock controls was used for expression studies in shake flasks. After one day of cell growth on BMGY medium at 30°C, 250 rpm, the BMGY medium was then switched to BMMY medium containing 0.5% methanol. The yeasts were fed with methanol every 24 h for a period of 96 h. The transformed strain, with the parental pPIC3.5K plasmid, was used as a control.

**Figure 1:**
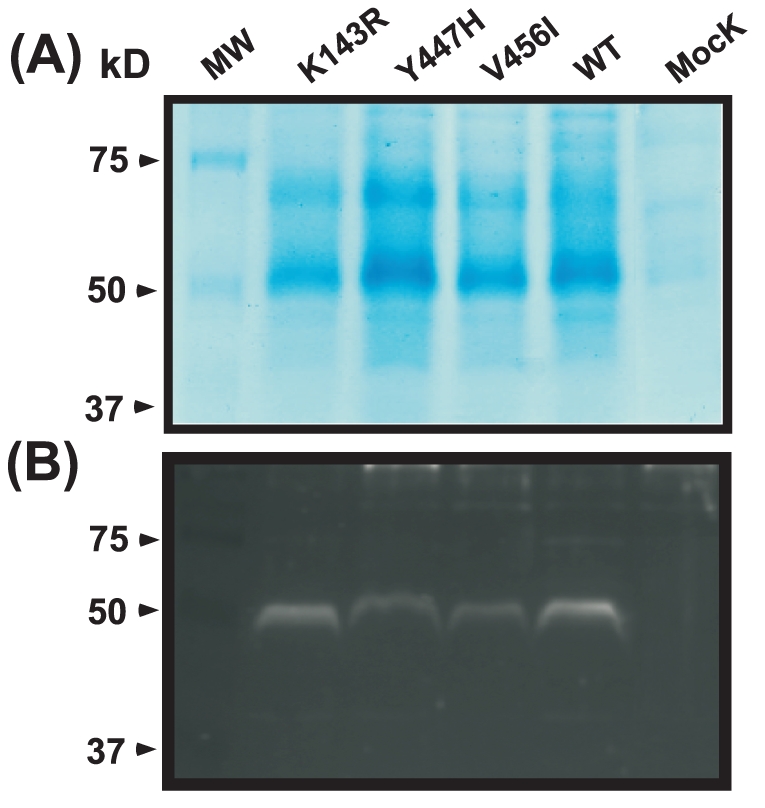
Expression of recombinant *Pichia pastoris* transformants. (A): SDS-polyacrylamide gel electrophoresis analysis. *P. pastoris* CaErg11p transformants were induced for protein expression on BMMY medium for 72 h as described in Material and Methods. Soluble cytosolic proteins were loaded into a 10% SDS-PAGE gel and stained with Coomassie brilliant blue. (B): Western blot analysis of control and mutated CaErg11p proteins produced in *P. pastoris*. Cytosolic proteins were transferred to PVDF membranes and incubated with a 1∶100 dilution of a polyclonal rabbit anti-yeast Erg11p and a 1∶2000 dilution of goat-anti rabbit-HRP. Signals were visualized using supersignal west pico substrate detection reagent.

SDS-PAGE shows that heterologous expression of the CaErg11p protein was successfully expressed into *P. pastoris*. After Coomassie gel staining, a band of around 56 kDa was detected in 0.5% methanol-induced clones in comparison to the mock control (Figure 1A). The overall levels of specific intracellular CaErg11p between *P. pastoris* mutants and wild-type control are likely to be relevant after Immunoblot with a polyclonal rabbit anti-*Candida* Erg11p analysis compared to the mock control (Figure. 1B). No band was detected in the induced strain transformed with the parental plasmid (Mock control). With respect to protein dosage, CaErg11p expression seems to be lower in Y447H and V456I mutants than wild-type control under the same experimental conditions.

### Azole susceptibility of *P. pastoris* CaErg11p transformants

**Figure 2:**
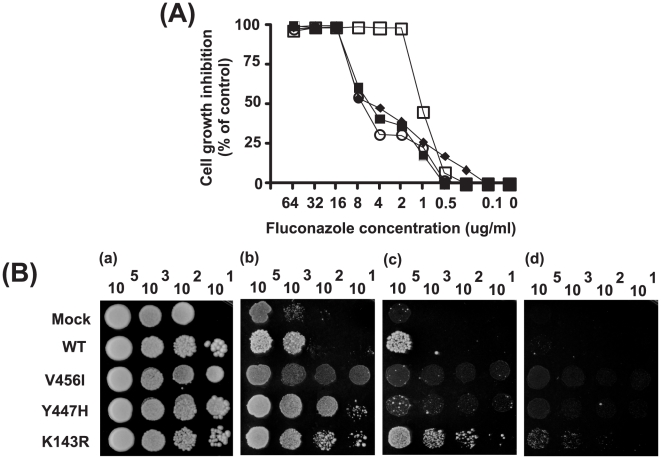
*In vitro* antifungal activity of FLC against *P. pastoris* transformants. (A): *P. pastoris* cells transformed with WT (□), K143R (▪), Y447H (○) and V456I (•) mutants of CaErg11p were tested according to the CLSI method with some modifications. MIC values were determined as the lowest antifungal concentration giving a 50% or less reduction in the optical density at 450 nm compared to the OD of the corresponding drug-free incubation medium. (B): Susceptibilities of CaErg11p *P. pastoris* transformants to azole fluconazole using spot assay. Serial dilutions of each control and mutant clones were spotted onto BMMY agar plates containing different concentrations of fluconazole and incubated for 72 h at 30°C. Untreated conditions (a), 2 µg/ml FLC (b), 4 µg/ml FLC (c) and 8 µg/ml FLC (d).

The susceptibility of *P. pastoris* transformants expressing the wild-type and mutant CaErg11p proteins to azole compounds was tested by a microdilution reference method with some modifications. The K143R substitution was used as resistance-positive control according to the site-directed mutagenesis studies showing that this substitution was responsible of a 64-fold increase of FLC MIC. *P. pastoris* transformants containing single mutations (Y447H, V456I or K143R) presented no difference in their MIC to FLC following growth on a non-inducing medium, BMGY (data not shown). CaErg11p was then induced in BMMY during 72 h. The susceptibility to azole compounds after induction was measured by modified-reference CLSI test with some modifications. Under these conditions, methanol was added to 96-well plates every 24 h. These experiments showed that growth and induction into a BMMY medium led to a significant increase of FLC MIC (Figure 2A). The MIC value for *P. pastoris* transformants containing the Y447H and -V456I substitutions was 8 µg/ml for FLC, similar to K143R positive-control mutant. Compared to the wild-type and the parental plasmid transformants, these values represented an 8-fold increase in FLC resistance. The MICs to VRC and ITR following growth and induction into a BMMY medium of *P. pastoris* CaErg11p transformants containing single substitutions were similar, indicating that Y447H and V456I mutations were specific for the FLC binding to CaErg11p.

The susceptibility of CaErg11p *P. pastoris* transformants to FLC was also qualitatively measured by spotting serial dilutions of each clone on BMMY medium containing various FLC concentrations (Figure 2B). Under these conditions, 0.5% methanol was added during CaErg11p pre- induction in BMMY and only at the beginning of spot tests. The wild-type and mock *P. pastoris* transformants were hypersensitive to FLC at 2 µg/ml, whereas the growth of Y447H, V456I and K143R *P. pastoris* mutants remained unchanged (b). After 72 h, *P. pastoris* mutants showed a better capacity to growth at high yeast dilution than controls when they were treated with 4 and 8 µg/ml FLC (c, d).

### FLC inhibition of ergosterol biosynthesis in *P. pastoris* CaErg11p transformants

**Figure 3:**
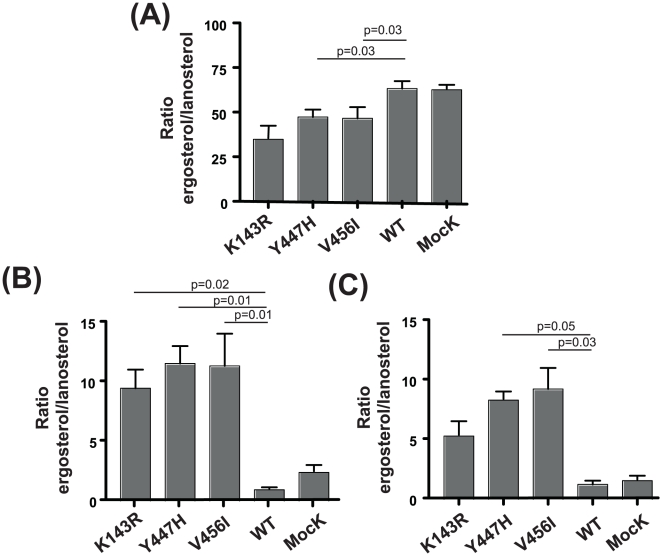
CaErg11p activity of *P. pastoris* transformants in the presence of FLC. *P. pastoris* CaErg11p methanol-induced transformants were treated with FLC in BMMY medium for 24 h at 30°C. Non-saponifiable lipids (sterols) were extracted as described in Material and Methods. Sterol identification was done in reference to the relative retention times and mass spectra previously reported [54], [55]. Activity results were expressed as the ratio of ergosterol biosynthesis compared to the lanosterol accumulation (E/L). (A): Untreated *P. pastoris* clones, (B): 4 µg/ml FLC and (C): 8 µg/ml FLC (n = 4).

The effect of single substitutions on fluconazole resistance was determined by measuring the catalytic activity of *P. pastoris* transformants CaErg11p through the analysis of yeast sterol composition by gas chromatography-mass spectrometry. The activity was expressed as the ratio of ergosterol biosynthesis compared to the toxic lanosterol accumulation (E/L). As expected, under untreated conditions i.e without FLC (Figure 3A), sterol composition of wild-type and mock *P. pastoris* transformants, showed ergosterol as the dominant sterol (median E/L ratio of 64 for each control). No significant difference was observed for K143R, Y447H and V456I *P. pastoris* mutants (median E/L ratio of 35, 48 and 47, respectively). Under un-treated conditions a significant difference in E/L ratio was observed between *P. pastoris* mutants and wild-type control. The controls and mutants also showed small quantities of the intermediates of the ergosterol biosynthesis pathway, such as zymosterol, fecosterol and episterol.

The wild-type, mock and all CaErg11p *P. pastoris* transformants were then treated with FLC at two concentrations around their FLC MICs. Wild-type and mock *P. pastoris* transformants showed a marked reduction of sterol 14alpha-demethylase activity after treatment with FLC (E/L ratio = 1) which concords with their susceptibility to FLC. In contrast, the control mutant K143R maintained enzymatic activity despite FLC treatment with 4 and 8 µg/ml (median E/L ratio of 9.5 and 5.4, respectively). Interestingly, demethylation activity of Y447H and V456I mutants was significantly higher than wild-type control after treatment with 4 µg/ml of FLC as reflected in the E/L ratio of 11.5 and 11 respectively (Figure 3B). When they were treated with 8 µg/ml FLC, the E/L ratio were also significantly higher than wild-type control (8.4 for Y447H mutant and 9.3 for V456I) (Figure 3C).

### CaCYP51 Insertion loop model and azole-resistant substitution mapping

**Figure 4:**
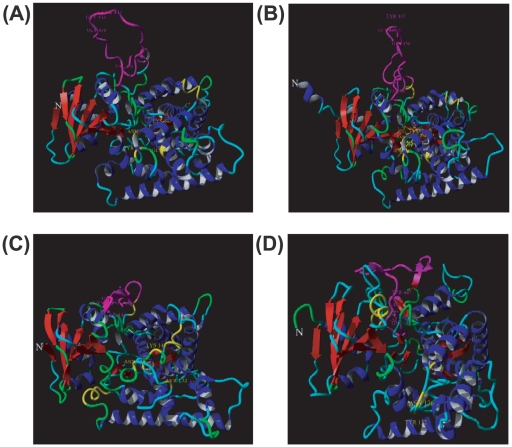
Localization of the major long insertion sequence of CaErg11p and azole-resistance substitutions mapping on CaErg11p structures obtained by homology modeling. (A): ModWeb model with human CYP51 ketoconazole liganded (3ld6). (B): YASARA model with human CYP51 liganded with econazole (3jus) with protoporphyrin IX and econazole represented as a stick respectively in orange and yellow. (C): SwissModel with *M. tuberculosis* 4-phenylimidazole liganded structure as template (1e9x). (D): ModWeb model zebrafish prostacyclin synthase CYP450 8a1 free (3b98). All the models are aligned, based on their secondary structure with the N-terminal part of the beta-strand rich domain left and the alpha-helices rich domain right. The secondary structure of the models are color-coded according to their type, red: beta-stands, blue =  alpha-helices, green =  turn, cyan  =  coil, magenta  =  the long fungi specific amino acid sequence insertion. Amino acid positions that have been proven to be responsible for azole-resistance are indicated with the amino acid name of the WT CaCYP51 with yellow for substrate binding site hot-spots and magenta in the insertion fungi specific sequence. N- and C-terminal are labeled by single letters N and C.

CaCYP51 (P10613) was modeled using 3 different homology modeling programs (Swiss-model, ModWeb and YASARA). All CaCYP51 structure models, obtained with the different programs, present the same overall fold typical of CYP51 proteins. The best model obtained by ModWeb and YASARA used a human CYP51, respectively ketoconazole (3ld6) and econazole (3jus) liganded versions presenting 45% sequence identity (residues 49–525). All the models differ essentially in the position in space of some connecting loops between alpha-helices of the all alpha-helical domain of CYP51 and in the position of the large insertion loop characteristic of fungi phyla CYP51 family members. ModWeb and YASARA modeled the sequence D_428_TAAAKANSVSFNSSDEVDYGFGKVSKGVSSP_459_ of CaCYP51 protein as a long coil pointing out from the protein core (Figure 4A and 4B). A small sheet, consisting of two small beta-strands, is located at the tip of the loop in the YASARA model. The last good Swiss-model represented this loop as a long 2 beta-strands hairpin-like structure with the DYGFG motif at the top (Figure 4C). This loop interacts with the alphaB helix and the loop containing the GGGRHR (alpha-K”) motif.

Interestingly, one of the two best ModWeb models using the CYP450 from zebrafish (3b98) represents this insertion with two small beta-stranded beta-sheet structures that exactly superpose CaCYP51 Y447 residue over the Y409 residue of the zebrafish version in close proximity to the catalytically important G_464_GGRHRC motif (Figures 4D and S4). Figure 4 localizes the azole-resistant amino acids positions mapped on the hybrid models. More than 140 amino acids substitutions of CaCYP51 were reported in literature (reviewed recently by Morio F. and coll. [29]. Using a criterion of reduced susceptibility isolates and azole-resistance which have been experimentally proven, we localized Y132F, Y132H, N136Y, K143R, G307S, S405F, Y447H, G448E, G448V, G450E and V456I. Three of these positions involved the substitution of a glycine in a bulkier amino acid, possibly demonstrating the importance of a reduced local flexibility for azole-resistance phenotype due to conformational restriction.

## Discussion

The understanding of the molecular mechanisms underlying the resistance to azole in *C. albicans* is essential, both in providing guidance in the selection of the appropriate antifungal agent at the onset of infection, and in the discovery of new antifungals which circumvent azole resistance. Intense research in recent years has highlighted azole resistance as a complex molecular process in which multiple mechanisms are involved, including overexpression of the efflux pumps genes [12], [20]–[22] and upregulation or substitution of the 14alpha-demethylase target enzyme CaErg11p [11], [23], [24]. Several of these mechanisms are frequently combined in a single clinical azole-resistant isolate. Sterol 14alpha-demethylase Erg11p encoded by the *Erg11* gene participates in ergosterol biosynthesis, a major sterol of the fungal cell membrane that is required for yeast viability, membrane fluidity and permeability [27]. From a medical perspective, Erg11p is the primary target of azole antifungal drugs. The reduction of azole affinity for Erg11p caused by amino acid substitutions is one of the most frequent mechanisms involved in azole resistance in *C. albicans* [28]. In a previous study, we identified two novel CaErg11p substitutions, Y447H and V456I possibly involved in azole resistance from two FLC-resistant (MIC = 64 µg/ml) clinical isolates (CAAL61 and CAAL37, respectively). These isolates presented other CaErg11p substitutions, G307S for CAAL61, and E266D, G464S, V488I for CAAL37 [29]. G307S and G464S have been previously identified in resistant clinical isolates [12]. G464S substitution has been confirmed to be the cause of FLC resistance in *C. albicans* due to reduced affinity for CaErg11p using site-directed mutagenesis [30]. G307S is often associated with other confirmed azole-resistant substitutions such as G450E and G464S.

In the first part of this study, we aimed to investigate the potential *in vitro* contribution of Y447H and V456I CaErg11p substitutions on FLC resistance. We used site-directed mutagenesis methodology of the wild-type *CaErg11* gene and heterologous expression into *P. pastoris*, in order to produce *P. pastoris* transformants expressing CaErg11p mutant proteins. We clearly demonstrated that both Y447H and V456I substitutions were responsible for an 8-fold increase in FLC MICs determined by a broth microdilution method when expressed into *P. pastoris* transformants compared to the wild-type and parental plasmid controls using CLSI tests. That resistance level was equivalent to the K143R positive control mutant. Next, we examined the activity of CaErg11p containing single substitutions, by measuring the sterol composition of *P. Pastoris* transformants before and after FLC treatment. Under un-treated conditions, we observed a significant difference between wild-type, mock controls and mutated *P. pastoris* transformants. This suggests that punctual mutations (K143R, Y447H and V456I) possibly have an effect on CaErg11p expression. This would be particularly relevant regarding the lower CaErg11p expression by westernblot compared to controls. In spite of the lowest ergosterol formation by mutant clones, these punctual mutations represents, however, an significant azole resistance advantage when they were treated by FLC compared to wild-type and mock controls. They showed a sustained capacity for producing ergosterol even in the presence of FLC. Seen together, these results clearly highlight the fact that Y447H and V456I are involved in fluconazole resistance confirming previous hypotheses with clinical isolates CAAL61 and CAAL37 [29].

Although, *Saccharomyces cerevisiae* is the most commonly employed model for studying the role of *C. albicans* Erg11p substitutions on azole resistance [31], [41], [42], we used in this study, the *P. pastoris* expression system as an alternative to the expression of mutated CaErg11p. Importantly, our results using *P. pastoris* displaying mutated CaErg11p with a modified broth microdilution method and sterol biosynthesis experiments, concord with the site-directed mutagenesis studies into *S. cerevisiae.* Moreover, *P. pastoris* has been proposed as an adapted expression system for mutagenesis studies [43]–[45]. Several elements supported these experiments: (i) the use of methanol inducible AOX1 promoter allows weak expression of native proteins; (ii) the selection of single copy-gene transformants avoids the over-expression of mutated proteins; (iii) *P. pastoris* leads to the production of post-translational modifications of mutated proteins such as glycosylation. Fourthly, in the area of directed evolution of CaErg11p by site-directed mutagenesis, the choice of an appropriate expression plasmid system is important in order to express and measure enzyme activity in their natural microsomal environment. Finally, *P. pastoris* allows the production and the purifying high levels of recombinant proteins for crystallographic and structural analyses by using extracellular expression plasmid systems.

Because all the fungal CYP51 that have been characterized were membrane bound microsomal proteins, their crystal structures have been difficult to resolve to date. The impact of CaCYP51 substitutions on interaction with azole antifungals was explained after 3D homology modelling using the amino acid sequence and the crystal structure of *Mycobacterium tuberculosis* as a reference [36]–[38]. Using this reference, the involvement of Y447H, V456I and the other amino acid substitutions located at the fungi-specific insertion sequence is difficult to question. Here, we proposed, for the first time ever, a novel hybrid model (YASARA) keeping the general overall core structure of the known CYP51 ortholog structures as described so far. This model helped us to map experimentally proved azole-resistant mutants (see Figure 4). The insertion sequence specifically found in fungal CYP51 orthologs was modeled as a long coil pointing out from the surface of the core protein. Because of the absence of homologous fungal CYP51 crystal structures, the position and interaction of this loop with the secondary structure core protein is difficult to model. In each of the 30 models obtained with YASARA, as well as the two models obtained by ModWeb and the swissmodel, the variable position of the insertion suggests its inherent flexibility. The insertion is localized between K' alpha-helix and the long L alpha-helix following the conserved catalytic C470 residue. Moreover, the insertion containing 39 possible positions in the recently published complete alignment of CYP51 family members [34] varies greatly in length. Depending on the fungal species, the insertion varies from 37 amino acid residues (*Pichia kudriavzevii*) to 27 amino acids (*Schizosaccharomyces pombe)*. *C. albicans* CYP51 having a 31 amino acid insertion. The half-N-terminal part of the insertion is variable in size and in sequence, unlike its half-C-terminal part which contains a short portion rich in acidic residues followed by an almost invariable sequence with the motif DYG[FY]Gx[VI][ST]KG. This motif is not shared with any protein, although it has a few similarities to a short sequence found in bacteria GTPase protein EngB.

Using the hybrid model, substitution Y447H is found to be solvent-accessible and is located in the middle of the invariable sequence portion of the insertion loop and could possibly modify the CaCYP51 local structure by disturbing the hydrogen binding network. Moreover, we could predict a possible pH influence of the mutant, because of the possible environmental dependence protonation of the H447 nitrogen atom of the azole ring. Moreover, the modeling of the CaCYP51 insertion sequence with the structurally similar sequence F_389_LNADRTEKKDFFKNGARVKY_409_PSVP_413_ found in zebrafish CYP450 structure (3b98) position, put the critical Y447 CaCYP51 in a position superposed over that of the Y409 zebrafish and in direct proximity to the catalytic important GGGRHRC motif. In contrast, the V456 position is located in the invariable sequence of the insertion loop. This position is mutated to isoleucine in almost 50% of the reported sequences in fungi phyla and could be described as a polymorphic position.

We note, with interest, that several other substitutions such as G448E, G448R, G448V, F449S, F449V and F449Y, previously associated with azole resistance, have been reported in this insertion region [15], [23], [24], [28], [47]. Moreover, protein sequence alignment of CaCYP51 with other fungal pathogens reveals that Y447 corresponds to Y431 in *Aspergillus fumigatus*, a residue (Y431C) involved in pan-azole resistance in this fungal species [48]. Looking at these models we could make a hypothesis of a loop-secondary structure substrate- or an azole-dependent conformation transition that could give better substrate availability or enzyme stability. Moreover, the flexibility of the region is probably enhanced by the relatively high glycine content occurring in the consensus sequence. Interestingly, the Y447H mutant is characterized by FLC resistance but remains highly susceptible to voriconazole which has the best Kd value for the wild-type CaCYP51 protein [49]. As previously hypothesized for I471T [49], FLC resistance in the Y447H mutant could be attributed to reduced affinity for its target.

The fact that amino acids at the insertion loop are conserved in several fungal species, probably indicates their implication in substrate-azole interaction. Clearer insights into the resistance mechanism of these mutants could be obtained by *in vitro* characterization of enzyme activities using purified wild-type and mutant proteins, recording circular dichroism and the ^1^H-NMR spectra of purified proteins in the presence of substrates/inhibitors in order to look for possible changes in secondary structure composition and by the elucidation of the *C. albicans* CYP51 crystallographic structure.

The construction of a heterologous expression system of CaErg11p mutants and a molecular model of clinical relevant amino acid substitutions highlight the potential role of the CaErg11p insertion loop in azole resistance. The rationale of preliminary knowledge of the resistance mechanisms before the design of new azole antifungal candidates, proposed by this comprehensive investigation, should be taken into consideration for future studies aimed at designing new azole antifungals against the human pathogen *C. albicans*.

## Materials and Methods

### Cells and vectors

*Escherichia coli* TOP10F' (Invitrogen, Inc.) competent cells were used for the transformation and propagation of recombinant plasmids. The pBluescript (SK-) (Stratagene GmbH) plasmid was used for *CaErg11* gene cloning and site-directed mutagenesis. The pPIC3.5K plasmid (Invitrogen) was used for sub-cloning and for the intracellular expression of CaErg11p protein mutants into the *P. pastoris* strain (KM71, *aox1::ARG4*, Mut^s^, His^−^). Mut designated «methanol utilization slow» phenotype was due to the loss of *AOX1* gene. The gene coding for CaErg11p was integrated behind the *AOX1* promoter.

### Reagents

Restriction enzymes BamHI, NotI, SacI, EcoRI, T4 ligase, calf intestinal phosphatase (CIP) and DNA molecular weight markers were purchased from New England Biolabs Inc. Phusion high fidelity DNA polymerase and buffers came from Finnzymes. GoTaq Flexi DNA polymerase was from Promega. Agarose was purchased from Invitrogen.

The NucleoSpin plasmid preparation kit and the NucleoSpin Extract II DNA fragment extraction kit from agarose gel were purchased from Macherey-Nagel GmbH. Acid washed beads, geneticin G418, ampicillin, kanamycin antibiotics, BCA assay reagents, methanol, n-hexane and glycerol and all oligonucleotide primers were obtained from Sigma Aldrich Co. Molecular weight marker for SDS-PAGE, electrophoretic transfer cell for western blotting and SDS-PAGE equipment were purchased from Biorad. A polyclonal anti-*Candida* Erg11p was kindly provided by Dr. Diane Kelly from the Institute of Life Science, School of Medecine, Swansea University, Swansea, UK. Anti-rabbit HRP conjugate was purchased from Cell Signaling Technology Inc. Super signal West Pico substrate solution was purchased from Thermo Scientific. FLC was purchased from Sigma, itraconazole (ITR) from Janssen-Cilag, and voriconazole (VRC) from Sigma Aldrich Co.

### *In vitro* and *in vivo* FLC susceptibility of *C. albicans* clinical isolates

Antifungal susceptibility to FLC, ITR and VRC was determined *in vitro* for CAAL37 and CAAL61 clinical isolates using the broth microdilution reference method, as recommended by the Clinical and Laboratory Standards Institute (CLSI) document M27-A2 [50]. MIC, that is the lowest drug concentration that resulted in 50% growth inhibition relative to the growth in the control well, was visually determined after 48 h of incubation at 35°C.

The *In vivo* antifungal activity of FLC for the CAAL37 and CAAL61 clinical isolates was evaluated in a mouse disseminated candidiasis infection model, according to a previous study [51] with the following modifications. CAAL97 isolate was used as a FLC susceptible control. Briefly, four-week-old female Swiss mice purchased from Elevages Janvier, were housed at the Experimental Therapy Unit (Faculty of Medicine, Nantes, France). Immunosuppression in mice was induced by subcutaneous administration of prednisolone (30 mg/kg; Sigma Aldrich Co) one day before infection. Disseminated candidiasis was induced by intravenous infection with 5×10^5^ *C. albicans* cells (CAAL37, CAAL61 and CAAL97 clinical isolates) in 0.1 ml suspension. Fluconazole (5 mg/kg) was then administered orally once a day, for 5 days, starting 1 h after infection. Virulence control groups were inoculated with *C. albicans* isolates and were treated with PBS as described above. Survival was monitored every day, for two weeks after infection. The survival rate was compared to the control group by using the logrank test and a *p* value of less than 0.05 was considered significant.

### Culture medium for bacteria and yeast

Luria broth medium supplemented with 100 µg/ml ampicillin was used for our bacterial culture. 10X YNB: 13.4% yeast nitrogen base with ammonium sulfate without amino acids. YPD medium: 1% yeast extract, 2% peptone, 2% dextrose. YPD-geneticin plates: the same as YPD plus 2% agar and variable amounts of geneticin. Minimal glycerol medium without histidine (MGY): 1.34% YNB, 1% glycerol, 4×10^−5^% biotin. Regeneration dextrose medium without histidine (RD): 1 M sorbitol, 2% dextrose, 1.34% YNB, 4×10^−5^% biotin, 0.005% amino acids. RDB plates: the same as RD with 2% agarose. Buffered glycerol-complex medium (BMGY): 1% yeast extract, 2% peptone, 100 mM potassium phosphate, pH 6.0, 1.34% YNB, 1% glycerol. Buffered-methanol complex medium (BMMY): the same as BMGY with 0.5% methanol replacing glycerol. Breaking buffer: 50 mM sodium phosphate, pH 7.4, 1 mM PMSF, 1 mM EDTA, 5% glycerol.

### Isolation and cloning of *CaErg11* gene into pBluescript(SK-)

The *CaErg11* gene was isolated from a wild-type *C. albicans* strain isolated from the Mycobank of the Parasitology and Medical Mycology Department, Nantes, FR. The ORFBamHI and ORFNotI primers containing a Kozak consensus sequence, were designed to amplify a 1745 bp cDNA fragment based on the reported sequence from the GeneBank nucleotide sequence database accession number X13296.1. PCR conditions were as follows: initial denaturation at 98°C for 5 min, followed by 35 cycles each at 98°C for 1 min, 58°C for 1 min and 72°C for 1 min. Finally one cycle was performed at 72°C for 10 min.

The *CaErg11* gene fragment and pBluescript(SK-) were prepared for restriction endonuclease digestion with BamHI and NotI. These primers facilitated the sub-cloning of the coding sequence into pPIC3.5K. The plasmid (10 µg) and cDNA fragment (1 µg) were mixed separately with 10 U/µg BamHI, 20 U/µg NotI, 5 µl 10X BSA, 5 µl 10X NEB3 buffer to a final volume of 50 µl. After incubation for 1 hour at 37°C, the digests were purified on 1% agarose gel by using the NucleoSpin Extract II kit (Macherey-Nagel). For cloning, 20 ng of digested pBluescript(SK-) and 60 ng of *CaErg11* cDNA fragment were ligated in 2 µl 10X ligase buffer (Invitrogen), 1 µl of T4 ligase (2000 U/ml, Invitrogen) and water to a final volume of 20 µl. The ligation mixture was incubated overnight at 16°C. Background ligation was determined by self-ligation of the plasmid and the circular plasmid was used as a positive control. The recombinant plasmid was named pBSSK-*CaErg11* and electroporated into TOP10F' *E.coli* cells.

### Site-directed mutagenesis

Site-directed mutagenesis was carried out using a QuikChange mutagenesis kit (Stratagene). The cloned *CaErg11p* gene (into pBlueScript(SK-)) was used as the starting material for constructing all the mutants. Mutagenic primer sequences for studying Y447H and V456I substitutions are presented in Table 2. The K143R substitution was selected as resistance-positive control according to the site-directed mutagenesis studies published by Chau et al., 2004 [15]. This substitution was responsible of a 64-fold increase of FLC MIC. Briefly, Phusion DNA polymerase was used to replicate recombinant plasmid strands with high fidelity. The mutagenic primers, which were complementary to the opposite strands of plasmid, were used to induce nucleotide mutations. DpnI endonuclease specific for methylated and hemimethylated DNA was used to digest the parental DNA template and to select for mutation-containing synthesized DNA. The nicked plasmids were then transformed into *E. coli* TOP10F' competent cells. The full *CaErg11* mutant coding region was sequenced using internal primers [52], [53].

**Table 2:** Oligonucleotide primers used in this study.

Primer	DNA sequence
ORFBamHI	5′-GCGGATCCACATATGGCTATTGTTGAAACTG-3′
ORFNotI	5′-TAGCGGCCGCTTAAAACATACAAGTTTCTCTTTT-3′
K143Rfor	5′-GGAACAAAGAAAGTTTGCTAAATTTGC-3′
K143Rrev	5′-GCAAATTTAGCAAACTTTCATTGTTCC-3′
Y447Hfor	5′-GATGAAGTTGATCATGGGTTTGGG-3′
Y447Hrev	5′-CCCAAACCCATAGTCAACTTCATC-3′
V456Ifor	5′-GTTTCTAAAGGGATTTCTTCACC-3′
V456Irev	5′-GGTGAAGAAATCCCTTTAGAAAC-3′
CaCYP51R2	5′-AATATAGTTGAGCAAATGAACG-3′
5′AOX1	5′-GACTGGTTCCAATTGACAAGC-3′
3′AOX1	5′-GCAAATGGCATTCTGACATCC-3′

### Construction of recombinant *Pichia pastoris* transformants

The general strategy of mutagenesis into *P. pastoris* is shown in Figure S2. The *CaErg11* gene was isolated from pBlueScript(SK-) by using BamHI and NotI enzymes and purified after agarose gel electrophoresis with a NucleoSpin Extract II kit. Purified fragment was subcloned into the *P. pastoris* intracellular expression plasmid pPIC3.5K. The recombinant clones were confirmed through PCR analysis with ORFBamHI and ORFNotI primers and sequencing (Figure S3.A).

The recombinant pPIC3.5K-*CaErg11* was used to transform *P. pastoris* KM71 strain by electroporation. Briefly, 10 µg of recombinant plasmids were linearized by 10 U/µg SacI leading to targeting plasmid for the *P. pastoris* chromosome at the *AOX1* locus. Linearized plasmid were purified from agarose and mixed with 80 µl competent *P. pastoris* cells. The mixture was transferred into an ice-cold 0.2 cm cuvette and an electric shock was given at 2 Kvolts for integration into the *P. pastoris* genome. Then, 1 M sorbitol was immediately added. The 0.6 ml of mixture was poured onto the top of RDB plates and incubated at 30°C until colonies appeared. The positive transformants that produced histidine were screened for the ability to grow on YPD-geneticin plates ranging from 0.25 mg/ml to 2 mg/ml geneticin. The yield of plasmid transformation was about 3000 colonies per transformation. Thirty colonies of pPIC3.5K-CaERG11-K143R, 27 colonies of pPIC3.5K-CaERG11-Y447H, 17 colonies of pPIC3.5K-CaERG11-V456I and 20 colonies of pPIC3.5K-CaERG11-wild-type were gathered from the YPD-geneticin plates at the 0.5 and 0.75 mg/ml concentrations, in order to test single-copy colonies. The His^+^Mut^s^ (methanol utilization slow) transformants were picked onto RDB plates and resuspended in YPD containing 15% glycerol and stored at −80°C.

### Analysis of *P. pastoris* recombinant transformants

The geneticin-resistant colonies were also grown in YPD for 24 h, then the genomic DNA was purified. PCR amplification of the *CaErg11* gene was carried out with 5′ AOX1 and 3′ AOX primers. Another PCR analysis was also done using 5′AOX1 and a *CaErg11* internal R2 primer. The PCR product of recombinant clones was a band at 1956 bp corresponding to the *CaErg11* gene (1745 bp) and a piece of pPIC3.5K plasmid (214 bp) (Figure S3.B). A band at 714 bp corresponding to a piece of AOX1 promoter (50 bp) and 664 bp of the 5′ extremity of the *CaErg11* gene indicates that all *P. pastoris* transformants were recombined (Figure S3.C). Recombinant *P. pastoris* with the parental plasmid was used as PCR control.

### Sequencing analysis

DNA sequencing was performed using an Applied Biosystems 3730 sequencer using internal primers [53]. Nucleotide sequences were assembled using Seqscape navigator software (Applied Biosystems). For each mutant, the entire *CaErg11* open reading frame sequence was compared to a previously described *CaErg11* sequence (accession number X13296). The *Erg11* sequences of the strains displaying new amino acid substitutions have been submitted to GenBank database under accession numbers EU885935 (CAAL37 isolate) and EU885936 (CAAL61 isolate). An exhaustive list of amino acid substitutions of each clinical isolate used in this study are presented in Table 1. The alignment data indicates that Y447H substitution was homozygous and V456I heterozygous.

### Small scale intracellular expression of recombinant CaErg11p proteins

*P. pastoris* cells transformed with wild-type, mock, K143R, Y447H and V456I mutants of the CaErg11p protein were analyzed for protein expression. Briefly, single colony transformants from RDB plates were inoculated into 1 ml of BMGY medium and incubated, whilst being agitated, at 30°C to reach an absorbance of 2 at 600 nm (18 h), then this 1 ml was inoculated into 10 ml of BMMY medium at 30°C. Incubation was prolonged for 96 h with the addition of methanol (0.5% v/v final concentration) every 24 h. Cells were collected every day by centrifugation at 3000 *g* for 10 min and analyzed for the expression level, protein activity and absorbance at 600 nm.

### Western blot analysis

For evaluation of the intracellular accumulation of proteins, cell pellets were resuspended in 100 µl ice-cold lysis buffer. Cells were mashed with 100 µl of glass beads (0.2 mm) by doing several 30 s vortex applications and a cooling cycle. Cell -mashed slurry was centrifuged at 800 *g* for 5 min at 4°C. The collected supernatants (soluble cytosolic proteins), were centrifuged at 13000 *g* for 10 min at 4°C. The protein content of clear supernatant was quantified using BCA assay. Forty µg protein per well were loaded into 10% SDS-PAGE gels. Electrophoresis was performed according to the Laemmli method. After electrophoresis, the gels were stained with Coomassie brillant blue R-250 and distained with a mixture of acetic acid-methanol-water (10∶25∶65 v/v). Proteins were also transferred to PVDF membranes and incubated with a 1∶100 dilution of polyclonal anti-*Candida* Erg11p produced in rabbit overnight at 4°C and a 1∶2000 dilution of goat-anti rabbit antibody coupled to HRP 1 h at room temperature. The signals were visualized using the super signal west pico substrate detection reagent.

### Azole susceptibility tests for *P. pastoris* transformants

The susceptibility of *P. pastoris* transformants expressing the wild-type and mutant CaErg11p proteins to azole compounds was tested using two methods. First, a broth microdilution reference method, as recommended by the Clinical and Laboratory Standards Institute (CLSI) document M27-A2 [50]. In some experiments RPMI1640 medium, which is usually used in this method, was replaced by BMMY liquid medium. A final concentration of 0.5% methanol at was added every 24 h to each well in order to guarantee protein induction. The MIC was defined as the antifungal concentration giving a 50% or less reduction in the optical density at 450 nm compared to the OD of the corresponding drug-free incubation medium. Secondly, a qualitative test was carried out by spotting serial dilutions of induced yeast onto agar plates containing BMMY medium. The azole compounds were diluted at different concentrations into BMMY plates. The *P. pastoris* transformants were induced at 30°C for 72 h in BMMY liquid medium and diluted to 2×10^7^ cells per ml in 0.9% NaCl. Five microliters of this suspension and 5 µl of serial dilutions of each yeast culture were spotted onto each type of plate. The plates were incubated for 72 h and 96 h at 30°C.

### FLC effect on ergosterol biosynthesis by *P. pastoris* CaErg11p transformants

After *P. pastoris* CaErg11p induction for 72 h at 30°C in BMMY medium, yeast cells were harvested and treated with various concentrations of FLC in BMMY medium for 24 h at 30°C. The treated cells were then harvested and resuspended in 3 ml of 60% (wt/v) KOH and saponified by heating at 80°C for 2 h. Non saponifiable lipids (sterols) were extracted from the saponified mixture, twice, with 2 ml of n-hexane pooled, and dried under nitrogen. The sterols were suspended in 100 µl of bis(trimethylsilyl) trifluoride for 30 min for silylation. The silyled sterols were analyzed by gas chromatography-mass spectrometry (Agilent Technologies). The sterol identification was done in reference to the relative retention times and mass spectra previously reported [54], [55] (n = 4).

### CaCYP51 homology modeling

CaCYP51 (P10613) was modeled using 3 different homology modeling programs. The Swiss-model repository already contains a CaCYP51 model using *Mycobacterium tuberculosis* 4-phenylimidazole liganded structure as a template (1e9x). 2 homology models were retrieved from the server version of modeler (ModWeb) with human CYP51 ketoconazole liganded (3ld6) and zebrafish prostacyclin synthase CYP450 8a1 free (3b98) as templates. Finally, the homology modeling module of the YASARA program (www.yasara.org) [56] using the latest CYP51 crystallographic structures that have been resolved, was used. YASARA homology modeling uses 6 different crystallographic structures as templates from different phyla, *M. tuberculosis* liganded (2w0b and 2w0a), human econazole liganded (2jus) and *T. brucei* liganded (3gw9) and free (3g1q), *T. cruzi* liganded (2wx2) selected automatically by PSI-BLAST optimized sequence alignments with the target sequence. After secondary structure prediction, loop construction and amino acid rotamers selection, molecular dynamic steps were performed with the YASARA default force field parameters and the 6 energetically lowest structures per template were retained after stereochemistry validation. A total of 30 models were then scored, sorted by Z-score and a unique hybrid model consisting of the best part of the 30 models was obtained. This hybrid model was submitted to a final molecular dynamics round and its quality evaluated and retained if its pertinence had improved. The effects of Y447H and V456I mutations at a molecular level were then further analyzed using the Foldx plugin within the program YASARA.

### Ethics Statement

This study was carried out in strict accordance with the recommendations of the Directive 86/609/EEC on the protection of animals used for experimentals and other scientific purposes. The protocol was approved by the Committee on Ethics of Animal Experiments of the Experimental Therapy Unit (UTE) of the Faculty of Medicine, University of Nantes, France (C-44015). The mice were anesthetized by inhalation of an isoflurane-air mix (2%, 1 l/min) before any manipulation and all efforts were made to minimize suffering.

## Supporting Information

Figure S1 FLC susceptibility of clinical isolates. The *In vivo* antifungal activity of FLC against the clinical isolates was evaluated in a mouse disseminated candidiasis infection model. Briefly, four-week-old female Swiss mice were immunosuppressed by subcutaneous administration of prednisolone (30 mg/kg) one day before infection. Disseminated candidiasis was induced by intravenous infection with 5×10^5^ *C. albicans* cells. After 1 h after infection, mice were treated either with PBS or FLC (5 mg/kg) orally once a day, for 5 days. Survival was monitored every day, for two weeks after infection. The survival rate was compared to the control group by using the logrank test and a *p* value of less than 0.05 was considered significant. CAAL37 (▪), CAAL37+ FLC (□), CAAL61 (▴), CAAL61+ FLC (▵), CAAL97 (•) and CAAL97+ FLC (○).
(EPS)

Figure S2 Outline of a strategy for cloning wild-ype and mutated *CaErg11* genes from *C. albicans* isolates and for predicting alterations of CaErg11p.
(EPS)

Figure S3 Construction of recombinant *Pichia pastoris* transformants. (A): Integration of CaErg11 mutant genes into mutagenesis pBSSK(-) plasmid confirmed by PCR using ORFBamHI and ORFNotI primers. Lane 1: pBSSK-CaErg11-K143R, lane 2: pBSSK-CaErg11-Y447H, lane 3: pBSSK-CaErg11-V456I, lane 4: pBSSK-CaErg11-wild-type and lane 5: PCR of pBSSK(-) mock control. (B): The genomic DNA from representative *P. pastoris* transformants was purified. PCR amplification of the *CaErg11* gene with 5′ AOX1 and 3′ AOX primers. (C): PCR amplification of the *CaErg11* gene with 5′AOX1 and a CaErg11p internal R2 primer. Lane 1: CaErg11-K143R, lane 2: CaErg11-Y447H, lane 3: CaErg11-V456I, lane 4: CaErg11-wild-type and lane 5: PCR of pPIC3.5K transformed *P. pastoris*.
(EPS)

Figure S4 ModWeb model of CaCYP51 (P10613) with zebrafish prostacyclin synthase CYP450 8a1 free (3b98) as template. The long fungi specific loop is represented in magenta. Mapped are the amino acid substitutions Y447H, G448E, G450E and V456I.
(EPS)
